# Low Toxicity of Metal-Organic Framework MOF-74(Co) Nano-Particles In Vitro and In Vivo

**DOI:** 10.3390/nano12193398

**Published:** 2022-09-28

**Authors:** Suke Lan, Jiahao Zhang, Xin Li, Lejie Pan, Juncheng Li, Xian Wu, Sheng-Tao Yang

**Affiliations:** 1 Key Laboratory of Pollution Control Chemistry and Environmental Functional Materials for Qinghai-Tibet Plateau of the National Ethnic Affairs Commission, School of Chemistry and Environment, Southwest Minzu University, Chengdu 610041, China; 2 Key Laboratory of General Chemistry of the National Ethnic Affairs Commission, School of Chemistry and Environment, Southwest Minzu University, Chengdu 610041, China

**Keywords:** metal-organic framework, nanoparticle, cell cycle arrest, biodistribution, oral toxicity

## Abstract

With the rapid development of metal-organic frameworks (MOF), the toxicity and environmental safety of MOF materials should be thoroughly investigated. The behaviors and bio-effects of MOF materials after oral exposure are largely unknown. In this study, we performed a pilot toxicity evaluation of MOF-74(Co) nanoparticles (NPs) both in vitro and in vivo. The cell viability and cell cycle were monitored after LO2 cells were incubated with MOF-74(Co). The Co contents, bodyweight, serum biochemistry, histopathological changes, and oxidative stress parameters were measured after oral exposure to MOF-74(Co) NPs in mice. LO2 cells showed viability loss at 100 mg/L. The cell cycle arrest was more sensitive, which was observed even at 12.5 mg/L. MOF-74(Co) NPs led to a significant accumulation of Co in the liver and kidneys. No bodyweight loss was observed and the serum biochemical index was mainly unchanged. Except for slight inflammation, the histopathological images of the liver and kidneys after oral exposure to MOF-74(Co) NPs were normal compared to the control. Meaningful oxidative stress was found in the liver and kidneys. The results collectively indicated the low toxicity of MOF-74(Co) NPs after oral exposure in mice.

## 1. Introduction

Metal-organic framework (MOF) materials are emerging materials with outstanding properties and wide applications. MOF materials are composed of metal cores and organic ligands that form a porous crystalline structure. Among these MOF materials, MOF-74 is a type of MOF material using 2, 5-dihydroxytelephthalic acid as the organic ligands. MOF-74 has shown great potential in various areas. For example, MOF-74(NiMg) adsorbed CO_2_ and supported Ni nanoparticles (NPs) for methanation [1]. MOF-74(Fe) adsorbed dimethyl phthalate efficiently and catalyzed the degradation by advanced oxidation processes [2]. ZIF-8@Pd@MOF-74 showed high performance in chemoselective hydrogenation reactions [3]. MOF-74(Co) microflower had a specific capacitance of 164.2 F/g at 0.5 A/g in a three-electrode system and 62.5 F/g at 1 A/g in an asymmetric supercapacitor device [4]. Ketoprofen loaded on MOF-74(Mg) could be applied to treat osteoporotic pain [5]. The controlled release of ketoprofen exceedingly reduced pain-related cytokines and increased the secretion of osteogenic cytokines and the expression of inflammatory factors. To ensure the safe application of MOF-74, the toxicity and environmental hazards of MOF-74 should be evaluated.

The results in the literature indicate the toxicity of MOF materials to various organisms. Our group aimed to reveal the environmental toxicity of MOF-199. MOF-199 is toxic to pea seedlings, nitrogen-fixing bacteria, *Escherichia coli,* and *Staphylococcus aureus* by releasing Cu^2+^ to arouse oxidative stress [6,7,8]. MOF-199 inhibits the root development of pea seedlings and affects biomass gain. Photosynthesis of leaves is significantly disturbed by MOF-199, suggesting the potential threat of MOF to the carbon cycle [6]. For bacteria, different strains show different tolerances to MOF-199. In particular, MOF-199 inhibits the nitrogen fixation activity of nitrogen-fixing bacteria by lowering the *nif*H gene levels and blocking energy metabolism [7]. MOF-199 is also toxic to white rot fungi, inducing biomass loss, structural changes, and oxidative stress [9]. MOF-199 inhibits the laccase activity of white rot fungi and affects their decomposition activity. Another MOF material of concern is the ZIF family. ZIF-67 induces more hemolysis of red blood cells than ZIF-8, which is attributed to the free radical generation by ZIF-67 [10]. ZIF-67 NPs show more impact on learning, memory, and the neuropeptide signaling pathway compared to the submicron-scale ones [11]. Submicron-scale ZIF-67 is less toxic compared to ZIF-67 NPs. ZIF-8 and ZIF-67 transform into zinc carbonate (ZIF-8) and Co_3_O_4_ (ZIF-67) under visible-light irradiation, which induces unsaturated fatty acid biosynthesis and metal ion-binding transcription changes in *Chlorella vulgaris*, enhances the toxicity of ZIF-67, and alleviates ZIF-8 toxicity [12]. MIL-160 and IRMOF-3 are reported to be toxic to cells [13,14]. Unfortunately, there is only one toxicity study on MOF-74 to date. In Zhu et al.’s study of MOF-74(Mg), MOF-74(Mg) induced proliferation inhibition and apoptosis in vitro [15]. In addition, MOF-74(Mg) led to serum biochemical changes and cardiotoxicity in vivo. However, the toxicity of MOF-74(Mg) was surprisingly high compared to other MOF materials. The ultrahigh toxicity of MOF-74(Mg) could hardly be understood considering Mg ions were nontoxic. In other studies, the toxicity of MOF materials was usually associated with the toxic heavy metal center. The hazards of MOF-74 to organisms can also be considered from algae inhibition applications. Fan et al. used MOF-74(Cu) to inhibit the growth of harmful cyanobacteria through the release of Cu^2^^+^ and oxidative damage [16]. Therefore, it is worthwhile to investigate the toxicity of MOF-74 with a toxic heavy metal center.

Oral exposure is a common pathway of MOF to organisms in both biomedical applications and toxicity evaluations. For instance, Javanbakht et al. reported that MOF-5 could deliver 5-fluorouracil through an oral dosage to release the drug in stomach acid [17]. Zhou et al. achieved the oral delivery of insulin by UiO-68-NH_2_ and the bioavailability reached 29.6% [18]. In these oral delivery studies, the porous structure and large surface area of MOF materials were utilized, but the corresponding hazards were not well addressed. The oral toxicity of MOF materials was investigated by Liu et al. and MIL-101 was found to be nontoxic to male and female mice at a 1000 mg/kg/day exposure for 28 days [19]. Oral exposure to MOF materials can result in different bio-effects compared to other exposure pathways. Thus, the oral toxicity of MOF-74 is worthy of investigation.

In this study, we investigated the in vitro and oral toxicity of MOF-74(Co) NPs. MOF-74(Co) NPs were synthesized using the precipitation method. The cell viability and cell cycle of LO2 cells were measured after incubation with MOF-74(Co) NPs at 0–100 mg/L. The Co contents in organs were quantified by inductively coupled plasma-mass spectrometry (ICP-MS) after oral exposure to MOF-74(Co) NPs at 0–200 mg/kg bodyweight. The bodyweight, serum biochemistry, and histopathology of mice were recorded at 1, 7, and 28 d. The oxidative stress parameters of glutathione (GSH) and malondialdehyde (MDA) were analyzed for the liver and kidneys. The implications of the safety assessments of MOF materials are discussed.

## 2. Materials and Methods

MOF-74(Co) NPs were prepared using the precipitation method following Manue et al.’s protocols [20]. The as-prepared MOF-74(Co) NPs were characterized by SEM (JSM-7500, JEOL, Tokyo, Japan) and XRD (XD-6, Purkinje General Instrument Co., Beijing, China). For the XRD measurements, the MOF-74(Co) sample was well ground and tableted with a glass slide. The auto-subtraction of the background was applied.

For the cytotoxicity evaluations, MOF-74(Co) NPs were dispersed in DMEM medium and added to LO2 cells in 96-well plates (2.5 × 10^3^ cells/well) at 0–100 mg/L. After 48 h incubation, 20 μL of MTT solution (5 mg/mL) was added to each well and incubated for another 24 h. Then, the supernatant was discarded and formazan salt was dissolved with 150 μL of DMSO for 15 min. The absorbance values were measured at 570 nm using a Spectra MAX M5 microplate spectrophotometer (Molecular Devices, San Francisco, CA, USA) to calculate the relative cell viability of LO2 cells. In another set of experiments, flow cytometry (FCM) was used to detect the influence of MOF-74(Co) NPs on the cell cycle distribution. LO2 cells were seeded in a 6-well plate at 3 × 10^5^ cells per well and incubated for 24 h. The cells were then treated with MOF-74(Co) NPs for 48 h. Cells were harvested and fixed with ice-cold 70% ethanol at 4 °C for 12 h. The ethanol was removed and the cells were washed with ice-cold PBS. After that, the cells were detached from the plate using trypsinization and collected by centrifugation at 1000 rpm for 5 min. The cells were re-suspended in binding buffer, stained by the reagents of the Cell Cycle Detection Kit (keygentec, KGA512), and analyzed by FACS following the manufacturer’s instructions (Jiangsu KeyGEN BioTECH Corp., Ltd., Nanjing, China).

For the oral toxicity assessments, the animal experiments were checked and approved by the Ethics Committee of Southwest Minzu University. The experiments were performed strictly in accordance with the Animal Care and Use Program Guidelines of Sichuan Province, China. ICR mice (20 g) were obtained from Dashuo Experimental Animal Co., Chengdu, China. The mice were raised in plastic cages (5 mice/cage) and randomly divided into groups of 5 mice each after the acclimation. The mice were orally dosed at 0, 50, 100, and 200 mg/kg bodyweight, where MOF-74(Co) NPs were dispersed in 0.2 mL of saline solution. After the exposure, the behaviors of the mice were observed daily and their bodyweight was measured at 1, 7, and 28 d before the sacrifice. The blood samples were collected to prepare serum for biomedical index analyses and then the mice were sacrificed by cervical dislocation. The organs, including the heart, liver, spleen, lung, kidneys, stomach, and intestine, were dissected for Co content measurements, histopathological observations, and oxidative stress measurements.

For the Co bioaccumulation assessment, the tissue samples were lyophilized, ground, weighed, and digested in a mixture of HNO_3_ and HClO_4_ (*v*/*v*: 5:1) on a microwave digestion system (APL-MD6M, APL Ople Instruments Co., Chengdu, China). For serum biochemistry analysis, the supernatants of blood samples were collected after the incubation at room temperature for 1 h and centrifugation at 3000 rpm for 10 min. The samples were sent to Chengdu Lilai Biotechnology Co., China, for the assays. For the histopathological observations, the liver and kidney samples were fixed with a 4% paraformaldehyde solution. The fixed samples were embedded in paraffin, thin-sectioned, and mounted on glass microscope slides using the standard histopathological techniques. The mounted sections were stained with HE for optical microscopy and recorded following standard protocols. For the oxidative stress assays, the samples were minced and homogenized in a 4 °C saline solution to obtain the homogenates (10% of weight/volume). The homogenates were centrifuged at 2000 rpm for 10 min to obtain the supernatants for the measurements of the protein concentrations, GSH, and MDA levels. The detailed protocols could be found on the official website of the manufacturer: http://www.njjcbio.com/ (accessed on 20 November 2020).

## 3. Results

### 3.1. Characterization of MOF-74(Co) NPs

**Figure 1:**
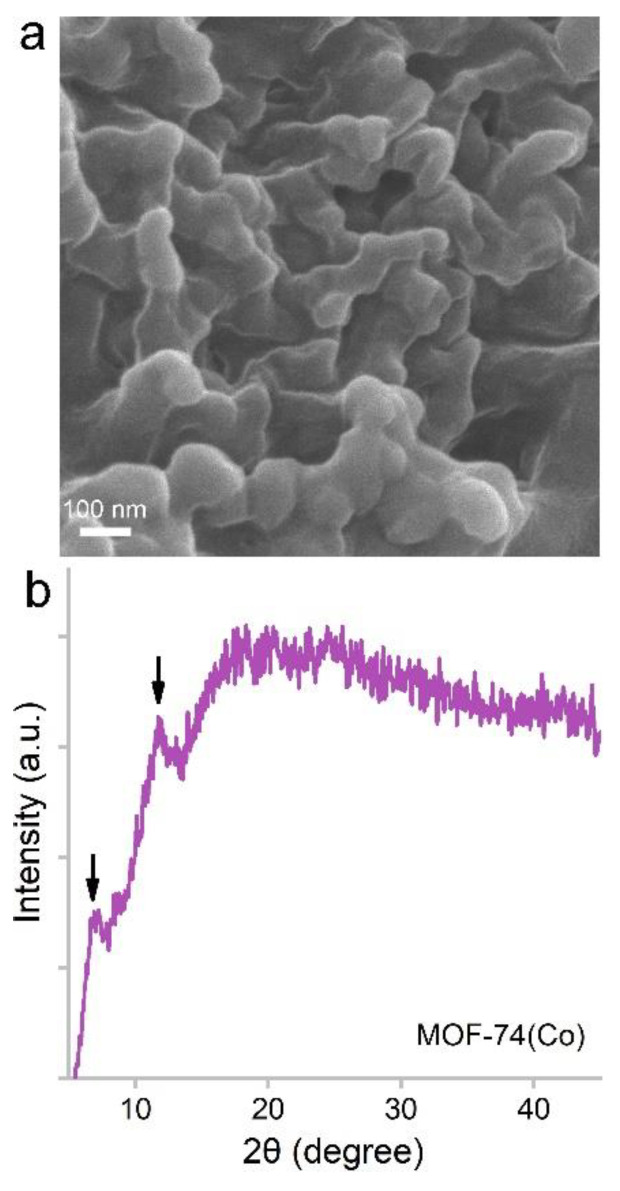
SEM image (**a**) and XRD spectrum (**b**) of MOF-74(Co) NPs. The arrows indicate the characteristic XRD peaks of MOF-74(Co) NPs.

MOF-74(Co) NPs were prepared using the precipitation method. Under a scanning electron microscope (SEM), MOF-74(Co) NPs were composed of small particles of about 50–100 nm in diameter (Figure 1). The particles aggregated with each other. The particle size and morphology were similar to the results in the literature [20]. The particle sizes of MOF-74(Co) were suitable for drug delivery applications, which could have enhanced the permeability and retention (EPR) effects [21]. The X-ray diffraction (XRD) spectrum of MOF-74(Co) showed two characteristic peaks at 7.03° and 11.90° (Figure 1), which was consistent with the literature [20]. For more detailed analyses, infrared spectroscopy, isotherm adsorption/desorption of N_2_, dynamic light scattering, and transmission electron microscopy would be able to provide more information on the functionalities, surface areas, porous structures, dispersion states, and particle sizes.

### 3.2. Cytotoxicity of MOF-74(Co) NPs

We selected the liver cell line (LO_2_ cell) as the model to investigate cytotoxicity because the Co bioaccumulation was mainly found in the liver. MOF-74(Co) NPs showed a concentration-dependent cell viability loss after 48 h treatment. The highest dose (100 mg/L) showed significant cytotoxicity with relative cell viability lower than 70% of the control. MOF-74(Co) NPs at lower concentrations from 12.5 mg/L to 50 mg/L did not affect the cell viability. The cytotoxicity was widely reported in previous studies of MOF materials. Wagner et al. compared the toxicity of MIL-160 and ZIF-8 to BEAS-2B cells and d H460 cells, where ZIF-8 was more toxic than MIL-160 [13]. IRMOF-3 was toxic to PC12 cells at 100 and 400 mg/L, inducing viability loss, membrane leakage, and improved differentiation [14]. ZIF-67 induced significant hemolysis of red blood cells [10]. MIL-100(Fe) showed dose-dependent toxicity to HL-7702 cells. Cell viability loss was detected at 20 mg/L and higher [22]. Membrane leakage, apoptosis, and necrosis were also identified. The EC_50_ of aminated ZIF-90 was 30 mg/L for HEK293 cells and MCF-7 cells [23]. MOF species are the most important due to their cytotoxicity. Tamames-Tabar et al. compared the IC_50_ of 14 types of MOF materials in J774 cells [24]. MIL-100 showed the highest IC_50_ of 700 mg/L and ZIF-8 had the lowest IC_50_ of 25 mg/L. The formation of the protein corona reduced the cytotoxicity of MOF materials. Human plasma incubation was reported to alleviate the viability loss induced by MOF-1 [25]. Compared to the results in the literature, the cytotoxicity of MOF-74(Co) NPs was moderate (Table 1).

**Figure 2:**
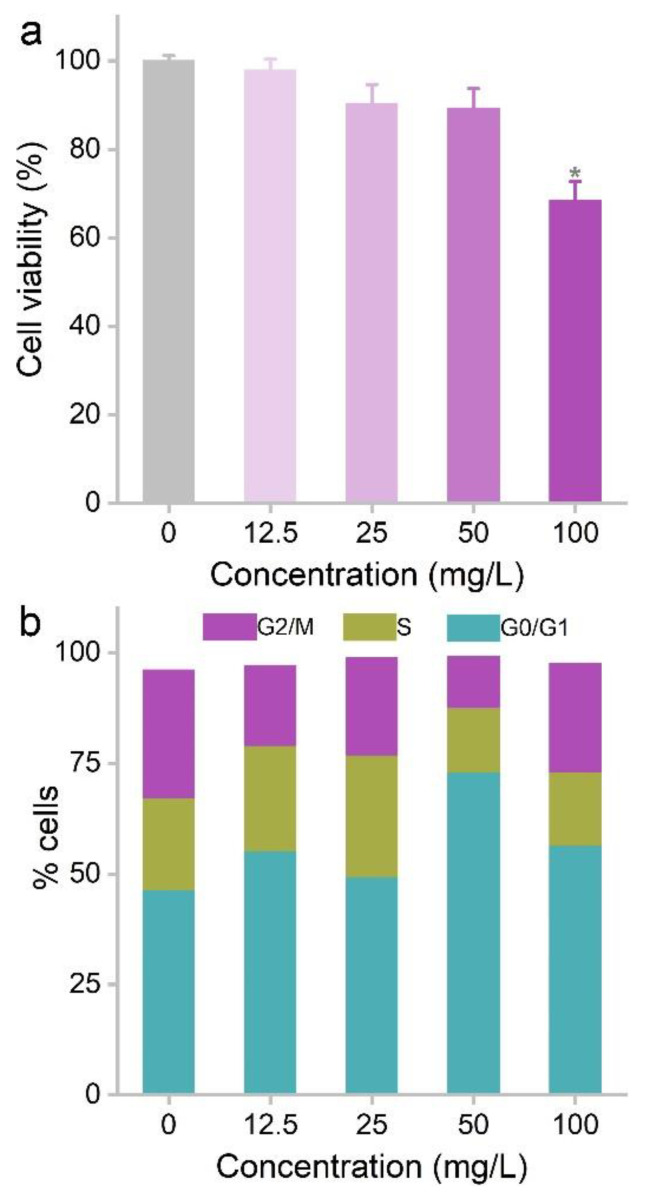
Influence of MOF-74(Co) NPs on the cell viability (*n* = 4) (**a**) and cell cycle (**b**) of LO2 cells. * *p* < 0.05 compared to the control group.

Cell cycle arrest seemed to be more sensitive to MOF-74(Co) toxicity. The LO2 cells were hindered at the G0/G1 phase even at 12.5 mg/L. The G0/G1 phase of the 50 mg/L group was 73.0%, which was much higher than that of the control group (41.6%). The cells were seriously damaged at 100 mg/L, which interfered with the cell cycle analysis, resulting in a decrease in the G0/G1 phase. The cell cycle arrest induced by MOF-74(Co) NPs led to the inhibition of cell proliferation, which was already reflected in the decrease in cell viability at 100 mg/L (Figure 2). Cell cycle arrest was also considered in the evaluations of other MOF materials. For example, Lin et al. reported the low cytotoxicity of ZJU-64 and ZJU-64-CH_3_ to PC12 cells [32]. Both MOF materials did not affect the cell viability and cell cycle distribution at concentrations up to 200 mg/L. Similarly, UiO-66_N did not change the cell cycle distribution [33]. ZIF-8 released Zn^2+^ to arouse oxidative stress and cell cycle arrest [34]. At 30 mg/L, the G2/M phase increased in 3T3 cells, HACAT cells, HEK cells, MB-231 cells, and MG-63 cells. However, no G2/M phase arrest was detected in RAW264.7 cells. Therefore, we suggested that cell cycle arrest could be used as a useful indicator for the cytotoxicity of MOF materials.

### 3.3. Biodistribution and Oral Toxicity of MOF-74(Co) NPs

**Figure 3:**
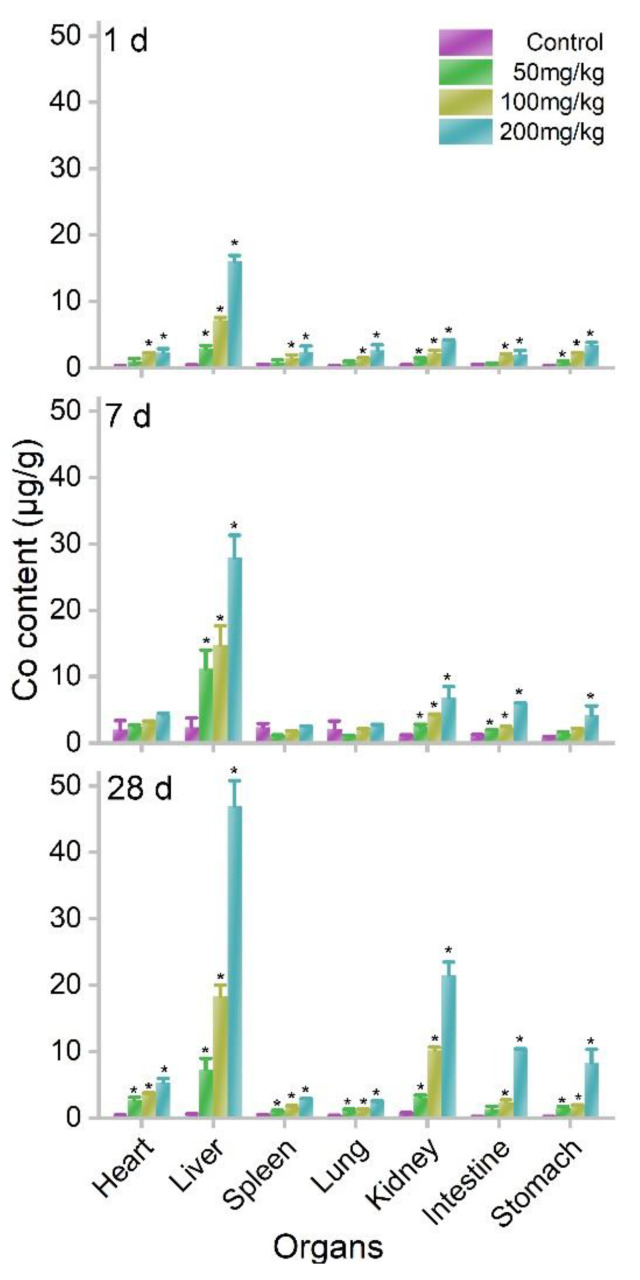
Co contents in organs after oral exposure to MOF-74(Co) NPs in mice at 1, 7, and 28 d (*n* = 3). * *p* < 0.05 compared to the control group.

The oral exposure of MOF-74(Co) NPs led to the accumulation of Co ions in the body because MOF-74(Co) NPs release Co ions in aqueous systems (55%). We quantified the biodistribution of Co in mice using inductively coupled plasma mass spectrometry (ICP-MS). The tissues were collected and digested for Co content measurements. The majority of Co was detected in the liver at 1 d postexposure. At 50 mg/kg bodyweight, the hepatic Co content was 2.93 μg/g. The Co accumulations were also significant in the kidneys and stomach. The kidneys had a Co content of 1.26 μg/g and the stomach had a lower Co content of 0.84 μg/g. At 100 and 200 mg/kg bodyweight, the accumulations in the heart, liver, spleen, lung, kidneys, intestine, and stomach were all statistically significant. The accumulation levels increased further at 7 and 28 d. In particular, the kidneys showed impressive increases in Co contents. At 200 mg/kg bodyweight, the Co content of kidneys was 4.16 μg/g at 1 d, which increased to 6.78 μg/g at 7 d and 21.54 μg/g at 28 d (Figure 3). This implied that Co started to be excreted through the urine pathway. The hepatic accumulation is a characteristic property of Co^2+^. Early in 1971, Smith et al. reported the distribution of ^60^CoCl_2_ in rats after gastric intubation and drinking water exposure [35]. The liver showed dominating Co accumulation in the first 2 months and Co was also detected in the skeleton, muscle, kidneys, pancreas, and spleen. The excretion pathways of Co ions were through urine and feces [36]. Beyond our study, the biodistribution of other MOF materials has been reported in the literature. MIL-100 NPs were quickly cleared from the bloodstream after intravenous injection [37]. MIL-100 NPs mainly accumulated in the liver (45.1% of injected dose) and spleen (4% of injected dose). Significant excretion of the ligand from urine was identified by high-performance liquid chromatography. Similarly, MIL-88A, MIL-100, and MIL-88B_4CH_3_ were reported to accumulate in the liver, spleen, and lung after intravenous injection, whereas the ligands were detected in urine [38]. Overall, the liver seemed to be the most important accumulating organ of MOF materials after intravenous injection, similar to other NPs [39]. The hepatic and splenic uptakes of NPs were due to the reticuloendothelial system (RES) capture, where NPs were surrounded by serum proteins and identified by the RES. On the other hand, after oral exposure, dissolved ions were absorbed by the intestine to enter the blood circulation. It is very unlikely that MOF-74(Co) NPs were directly bioaccumulated since MOF-74(Co) NPs were poorly dispersed and precipitated quickly. The liver as the most important organ in metabolism captured these toxic heavy metal ions. Therefore, hepatic accumulations of MOF NPs were observed after different exposures. The kidneys could play a crucial role in the excretion of MOF materials. However, the increases in bodyweight suggested that the excretion efficacy of MOF NPs was low. The slow excretion was a widely reported phenomenon of NPs and also heavy metal ions [39].

**Figure 4:**
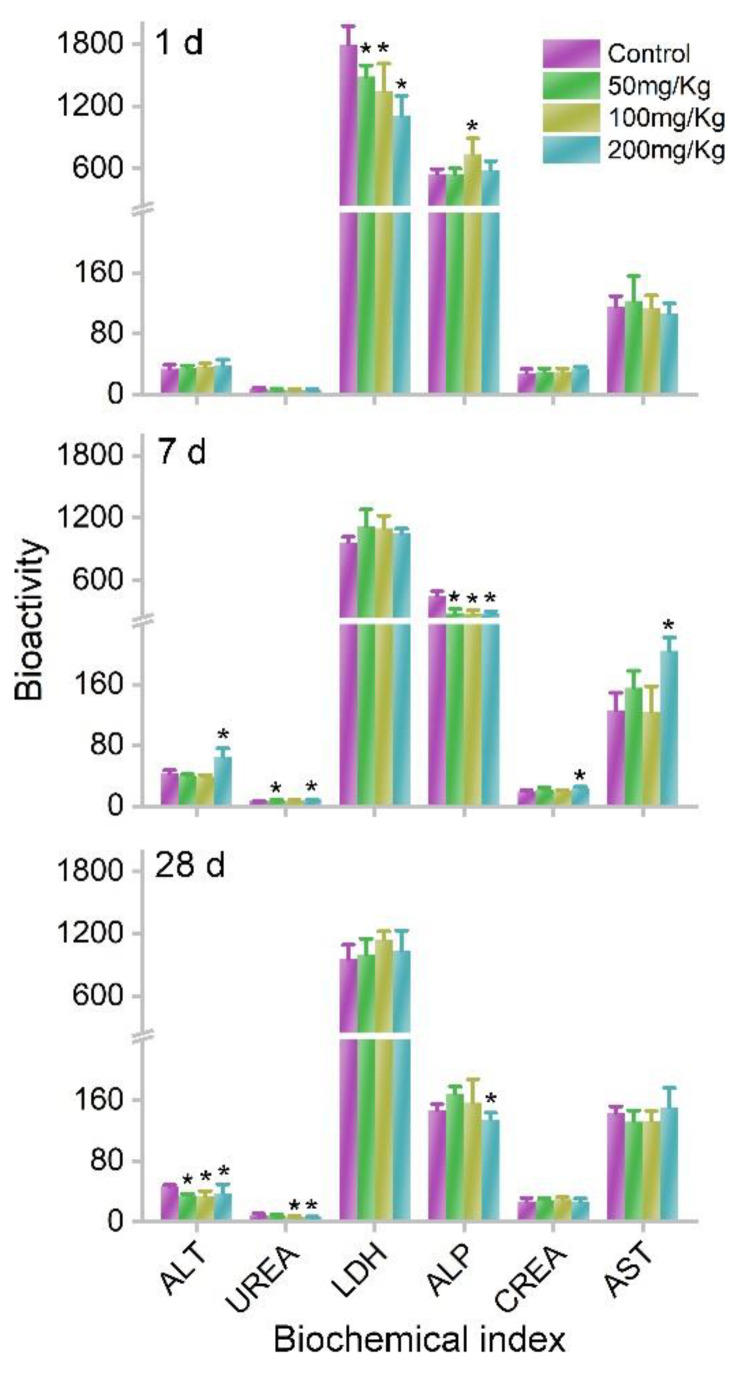
Serum biochemistry of mice after exposure to MOF-74(Co) (*n* = 4). * *p* < 0.05 compared to the control group.

Although meaningful Co accumulations were observed in the body, the bodyweight increases in mice were not affected by MOF-74(Co) NPs. The bodyweights were similar between the control group and MOF-74(Co) NP groups. The bodyweight increases were also similar between the control group and MOF-74(Co) NP groups. The serum biochemical analyses also indicated the low toxicity of MOF-74(Co) NPs after oral dosage. At 1 d, the lactate dehydrogenase (LDH) levels decreased after exposure to MOF-74(Co) NPs. A decrease in LDH usually suggests endocrine dyscrasia or burnout. Alkaline phosphatase (ALP) levels increased at 100 mg/kg bodyweight from 550 to 737. The ALP increase suggested that the possible inflammation occurred in the liver. The remaining parameters were all similar to those of the control group. At 7 d, the alanine aminotransferase (ALT) and aspartate aminotransferase (AST) levels increased significantly at 200 mg/kg bodyweight, indicating the hepatic toxicity of MOF-74(Co) NPs. The ALT and AST increases indicated that functional damage occurred in the liver. However, ALP levels decreased, implying the disappearance of inflammation. The serum urea (UREA) and creatinine (CREA) levels also increased at 7 d. This suggested that renal function was altered. No parameters increased at 28 d, which implied that the hepatic damage recovered (Figure 4).

**Figure 5:**
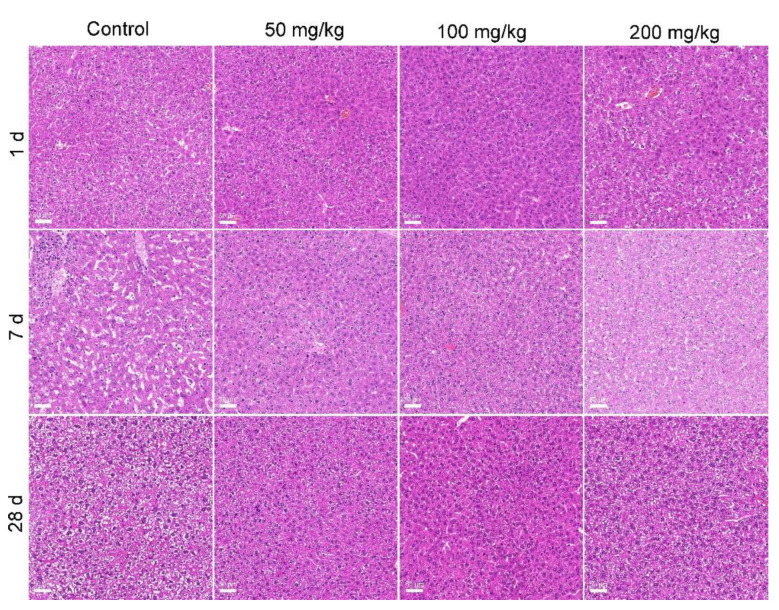
Histopathological observations of the liver after exposure to MOF-74(Co). The scale bar represents 50 μm.

**Figure 6:**
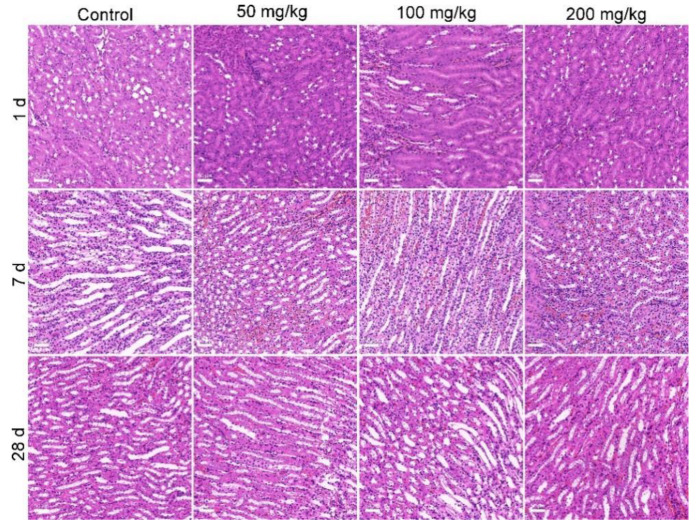
Histopathological observations of kidneys after exposure to MOF-74(Co). The scale bar represents 50 μm.

The histopathological observations supported the conclusion that MOF-74(Co) NPs were of low toxicity in vivo. The typical structure of the liver presented in the control and also the MOF-74(Co) NP groups, with normal hepatic cells and some red blood cells in the hepatic sinusoids (Figure 5). The normal histopathological characteristics of the liver suggested that although the hepatic function and inflammation might be altered by MOF-74(Co) NPs, there was no organic damage to the liver. In the histopathology of the kidneys, hyperemia was observed at 7 d after oral exposure to MOF-74(Co) NPs, which presented as pink cells (Figure 6). The symptom disappeared at 28 d. The phenomena were consistent with the serum biochemical changes, where renal toxicity was indicated by UREA and CREA at 7 d and renal function recovered at 28 d. The low toxicity of MOF-74(Co) NPs can be attributed to three factors. First, the bioavailability of MOF-74(Co) NPs was low. About 1.05–1.73% of the injected dose was detected in the body at 28 d. Lower bioavailability meant a less meaningful exposure of organs to pollutants, resulting in fewer hazards. Second, about half of the Co was released in aqueous systems. In our evaluation, only 55% of Co was detected in the supernatant after 24 h incubation ex vivo. Third, the low accumulation of Co was tolerable for the body. The toxicity of Co ions requires very long-term exposure. Domingo et al. reported that daily exposure to CoCl_2_ at 3.5–30.2 mg/kg bodyweight for 3–7 months did not change the hepatic histopathology of rats [40]. Oral exposure to CoCl_2_ at 4.5 mg/kg bodyweight for 5.5–8 months daily led to kidney damage [41]. In our study, mice were exposed to Co-containing MOF-74(Co) NPs at 50–200 mg/kg for only one dose. Thus, this was unlikely to exhibit the Co toxicity that only occurred in multi-dosages. In the literature, the in vivo toxicity of MOF materials has been seldom evaluated. Liu et al. reported that repeated oral dosages of MIL-101 for 28 d did not induce apparent acute and subacute toxicity at 1000 mg/kg/d [19]. MIL-100 induced significant increases in AST levels after intravenous injection in rats but ALT levels were not affected [36]. MIL-88A, MIL-100, or MIL-88B_4CH_3_ changed the ALT and AST levels, indicating that they serve hepatic toxicity [38]. ZIF-67 impacted learning, memory, and the neuropeptide signaling pathway in vivo [11]. In another study of intraperitoneal exposure to MOF-74(Mg), serum biochemical changes and cardiotoxicity were found at a dosage of 1000 mg/L × 100 μL, equaling about 0.59 mg/kg bodyweight [15]. MOF-74(Mg) was highly toxic compared to our results and other results in the literature, in particular considering that Mg ions are nontoxic and the particulate forms of MOF materials are generally less toxic than their metal ion centers [9]. In the future, the role of the metal ion center in MOF toxicity should be directly investigated and compared.

**Figure 7:**
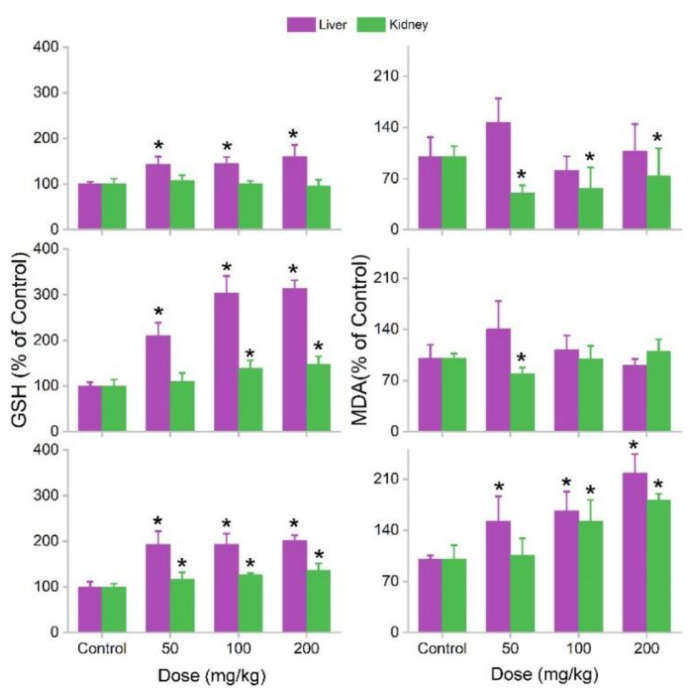
Oxidative stress of liver and kidneys after exposure to MOF-74(Co) (*n* = 4). * *p* < 0.05 compared to the control group.

Oxidative stress is a widely observed phenomenon in nanosafety assessments. GSH is a reductant reagent for scavenging oxidative radicals. MDA reflects lipid peroxidation. An increase in GSH and a decrease in MDA levels usually indicate oxidative stress, where the protection mechanism against oxidative damage is activated. When the stress is too serious and oxidative damage occurs, GSH is depleted and MDA accumulates. MOF-74(Co) NPs induced the GSH increases in the liver at 1 d. In the kidneys, the MDA level decreased. The changes in GSH and MDA indicated the stimulation of oxidative stress by MOF-74(Co) NPs and could be regarded as a defensive response to xenobiotics. At 7d, the increases in GSH in the liver were found to be greater and slight increases in GSH were found in the kidneys at 100 and 200 mg/kg bodyweight. The MDA decrease was only significant at 50 mg/kg bodyweight. At 28 d, the GSH increases were all significant for the liver and kidneys. Surprisingly, there were also meaningful increases in the MDA levels, indicating oxidative damage to the liver and kidneys (Figure 7). The oxidative stress induced by MOF-74(Co) NPs could be attributed to two contributors. First, the released Co ions were good catalysts for Fenton-like reactions [34]. In the Fenton-like reactions, hydroxyl radicals and other ROS were formed to attack cells and induce oxidative stress and/or damage. Second, the remnant MOF-74(Co) NPs may mechanically interact with tissues and mechanical damage could occur at the cellular level. Cell damage could arouse oxidative stress. Co^2+^-aroused oxidative stress has been reported in the literature. Mao et al. found that Co(II) catalyzed the decomposition of H_2_O_2_ to generate reactive oxygen species (ROS), which led to DNA damage and 20 -deoxyguanosine hydroxylation [42]. CoCl_2_ induced MDA increases in rats after acute and chronic exposures [43]. A decrease in GSH in acute exposure and an increase in GSH in chronic exposure were identified. Oxidative stress was also widely reported in MOF toxicity studies. We showed that MOF-199 induced oxidative stress in plants [6], bacteria [7,8], and white rot fungi [9]. Fe(III)-MOF accumulated in the RES after intravenous injection without toxicity, but it aroused significant oxidative stress [38]. Baati et al. also observed oxidative stress after intravenous injection of MIL-88A, MIL-100, or MIL-88B_4CH_3_ [37]. Thus, we suggest that oxidative stress widely exists for MOF toxicity. In our study, the oxidative stress aroused by MOF-74(Co) did not lead to significant toxicity in mice. In the future, the treatment of antioxidants should be investigated as a potential approach to alleviate the oxidative stress and toxicity of MOF-74(Co) NPs.

## 4. Conclusions

In summary, the nanosafety of MOF-74(Co) NPs was evaluated in vitro and in vivo, where MOF-74(Co) NPs showed low toxicity to LO2 cells and mice. MOF-74(Co) NPs induced cell viability loss at 100 mg/L, whereas cell cycle arrest occurred at 12.5 mg/L and higher. MOF-74(Co) NPs released Co in an aqueous system and led to meaningful Co accumulation in the liver and kidneys after oral exposure. No toxicity symptoms were observed, except for oxidative stress. Our results collectively indicated the low toxicity of MOF-74(Co) NPs. We hope that our results benefit the ongoing toxicity and environmental safety evaluations of MOF nanomaterials.

## Figures and Tables

**Table 1:** Cytotoxicity of different MOF materials.

MOF Material	Cell Line	Dose Range	IC_50_	Ref.
MIL-160	BEAS-2B	1–750 mg/L	421 mg/L	[13]
Fe-MIL-101	SKOV3	1.56–50 mg/L	23.6 mg/L	[26]
MOF-Zn_2_(1,4-bdc)_2_(dabco)	HuH7	0.01–10,000 mg/L	1000 mg/L	[27]
UiO-66	HeLa	10–100 mg/L	>100 mg/L	[28]
IRMOF-3	PC12	25–400 mg/L	100 mg/L	[14]
rMOF-FA	HeLa	0–120 mg/L	43 mg/L	[29]
Cu-BTC	MCF7	0–75 mg/L	3.49 mg/L	[30]
	HT-29		>25 mg/L	
	HL-60		>25 mg/L	
	NCI-H292		>25 mg/L	
Cu-MOF	MCF-7	0–200 mg/L	109 mg/L	[31]
MOF-74(Co)	LO2 cells	0–100 mg/L	>100 mg/L	This study

## Data Availability

The data presented in this study are available on request from the corresponding author.
